# The role of secondary outcomes in multivariate meta‐analysis

**DOI:** 10.1111/rssc.12274

**Published:** 2018-03-23

**Authors:** John B. Copas, Dan Jackson, Ian R. White, Richard D. Riley

**Affiliations:** ^1^ University of Warwick Coventry UK; ^2^ University of Cambridge UK; ^3^ University College London UK; ^4^ University of Keele UK

**Keywords:** Borrowing of strength, Multiple outcomes, Multivariate meta‐analysis

## Abstract

Univariate meta‐analysis concerns a single outcome of interest measured across a number of independent studies. However, many research studies will have also measured secondary outcomes. Multivariate meta‐analysis allows us to take these secondary outcomes into account and can also include studies where the primary outcome is missing. We define the efficiency *E* as the variance of the overall estimate from a multivariate meta‐analysis relative to the variance of the overall estimate from a univariate meta‐analysis. The extra information gained from a multivariate meta‐analysis of *n* studies is then similar to the extra information gained if a univariate meta‐analysis of the primary effect had a further *n*(1−*E*)/*E* studies. The variance contribution of a study's secondary outcomes (its borrowing of strength) can be thought of as a contrast between the variance matrix of the outcomes in that study and the set of variance matrices of all the studies in the meta‐analysis. In the bivariate case this is given a simple graphical interpretation as the *borrowing‐of‐strength plot*. We discuss how these findings can also be used in the context of random‐effects meta‐analysis. Our discussion is motivated by a published meta‐analysis of 10 antihypertension clinical trials.

## 1 Introduction

Univariate meta‐analysis is well established as a statistical tool for research synthesis, when a single outcome of primary interest is measured across several independent studies. Many research studies, however, report data on multiple outcomes, with the primary outcome supported by measures of one or more secondary outcomes. Multivariate meta‐analysis offers the potential for more accurate estimation by also taking the data on these secondary outcomes into account. Another advantage of the multivariate approach is the potential for increasing the number of eligible research studies, since we can also include studies where the primary outcome is missing and data are reported on only some of the secondary outcomes.

A key question in the expanding literature on multivariate meta‐analysis is the comparison between multivariate and univariate approaches—how much borrowing of strength do the secondary outcomes contribute to the estimation of the primary treatment effect? The empirical examples that were discussed by Sohn (2000), Simel and Bossuy (2009) and Trikalinos *et al*. (2014) mostly showed rather little difference between the results of multivariate and univariate meta‐analysis, even though in some of these examples the outcomes are quite highly correlated. This has led some to question whether the multivariate approach is of any real practical value. Other examples, however, suggest that taking the secondary outcomes into account can make a useful contribution (Fibrinogen Studies Collaboration, 2009; Riley, 2009; Kirkham *et al*., 2012). Why do these differences arise? What is it about the statistical properties of the studies in a meta‐analysis that determine the contribution of the secondary outcomes?

By comparing the multivariate estimate of the primary treatment effect with the corresponding univariate estimate taking only the primary outcomes into account, Jackson *et al*. (2017) derived an expression for borrowing of strength, measuring the additional contribution which each study's secondary outcome estimates make to the variance of the summary primary treatment effect on top of the contribution of the study's primary outcome estimate. The corresponding expression for the total contribution of individual studies gives a measure of study weights, analogous to the familiar use of study weights in univariate meta‐analysis. The aim of this paper is to re‐examine Jackson's formulae, to explore some of their consequences and extensions and to offer a more transparent understanding of how borrowing of strength depends on individual study characteristics. We generalize a number of points which earlier references have made by using examples and simulation studies. Data from a published meta‐analysis of 10 clinical trials on the treatment of hypertension is taken as a motivating example.

Section 2 gives our basic set‐up, showing that the borrowing of strength that is given by the secondary outcomes of the *i*th study can be written as an explicit function of two variance matrices: the within‐study variance matrix *V*_*i*_ and the harmonic average $\overset{¯}{V}$of all the *V*_*i*_s in the meta‐analysis. With an appropriate redefining of *V*_*i*_ (Section 2.2) this also covers cases where one or more of the outcomes in the *i*th study is missing. Properties of the borrowing‐of‐strength function are most easily seen in the bivariate case, where the *borrowing‐of‐strength plot* is a useful way of interpreting the relative contributions of the two outcomes. The bivariate case is discussed in Section 3 and illustrated by using data from the hypertension example. The bivariate case is generalized to the multivariate case in Section 4, leading to a general formulation of the necessary and sufficient conditions for a study to give borrowing of strength in multivariate meta‐analysis.

Section 5 follows Jackson *et al*. (2017) by showing that, at least as descriptive measures, borrowing of strength in multivariate fixed effects models applies equally well to random‐effects models, thus allowing for between‐studies heterogeneity in a way that is analogous to the DerSimonian–Laird (DL) method in univariate meta‐analysis (DerSimonian and Laird, 1986). A simulation study based on the hypertension example shows the importance of distinguishing between borrowing of strength as a descriptive measure (describing the data to hand) and as an inferential measure (describing an underlying population model): a distinction which does not arise in the same way for fixed effects models.

The final Section 6 gives a brief discussion of some of the important assumptions that are made in this paper.

## 2 The variance contribution of individual studies

### 2.1 Basic set‐up

We consider a multivariate meta‐analysis of *n* independent studies, each of which measures a *p*×1 vector *y* of treatment effect estimates corresponding to the *p* different outcomes. The standard multivariate fixed effects model is

(1) $$y_{i}∼N\left(\beta ,V_{i}\right),i=1,2,\ldots ,n.$$

The *p*×*p* variance matrix *V*_*i*_ in model (1) is specific to each study, but the unknown mean parameter *β* is assumed to be the same for all studies (the fixed effects assumption). To start with, we assume that all *p* outcomes are measured in all *n* studies in the meta‐analysis.

Treating each *V*_*i*_ as known (the usual assumption), the score function for the unknown parameter *β* (the derivative of the log‐likelihood) is

(2) $$\sum V_{i}^{-1}\left(y_{i}-\beta \right),$$

and so the maximum likelihood estimate of vector *β* is

(3) $$\hat{\beta }=\Omega \sum V_{i}^{-1}y_{i},$$

where Ω is the variance matrix of $\hat{\beta }$ given by

$$\Omega =\text{var}\left(\hat{\beta }\right)={\left(\sum V_{i}^{-1}\right)}^{-1}.$$

This can be rewritten as

$$\Omega =\frac{1}{n}\overset{¯}{V},$$

where

(4) $$\overset{¯}{V}={\left(n^{-1}\sum V_{i}^{-1}\right)}^{-1},$$

the harmonic average of the *V*_*i*_s. Whether we use the actual within‐study variances *V*_*i*_, or crudely approximate them all by $\overset{¯}{V}$, we end up with the same variance matrix of $\hat{\beta }$.

Even if all *p* components of *y* are observed, we focus interest on estimating the treatment effect for just one of these outcomes which, without loss of generality, we take to be the first. So from now on we shall describe, for each study, *y*_*i*1_ as the scalar treatment effect estimate for the *primary* outcome and the remaining components of *y*_*i*_ as the (*p*−1)×1 vector of estimates for the *secondary* outcomes. In some cases the primary outcome may be clearly identified from the context. For example, the bivariate (*p*=2) example in Fibrinogen Studies Collaboration (2009) concerned study estimates *y*_*i*1_ of a treatment effect adjusted for differences across a defined set of covariates, but it also included estimates *y*_*i*2_ which are partially adjusted for just a subset of these covariates. The fully adjusted results are of primary interest, but the advantage of including the secondary outcomes is that we can also take account of studies which do not measure the full set of confounding covariates. In other cases, such as the bivariate example that is studied in Section 3.2, we may be interested in all the outcomes, in which case we can arbitrarily relabel the outcomes as appropriate. The essential assumption is that we are interested in the separate (marginal) inferences to be made for one or more of the outcomes rather than in the correlations between the meta‐analysis estimates across different outcomes. So we assume from now on that our primary interest is in ${\hat{\beta }}_{1}$, with variance

$$\text{var}\left({\hat{\beta }}_{1}\right)=\Omega_{11}={\left[{\left(\sum V_{i}^{-1}\right)}^{-1}\right]}_{11}=\frac{1}{n}{\overset{¯}{V}}_{11},$$

where ${\overset{¯}{V}}_{11}$ is the (1,1) element of $\overset{¯}{V}$ in equation (4).

A natural comparison for the multivariate estimate of *β*_1_ is univariate meta‐analysis, which looks only at the values of *y*_1*i*_ and ignores the data on the secondary outcomes. The relevant univariate model would then be

$$y_{i1}∼N\left(\beta_{1},\sigma_{i}^{2}\right),$$

where

$$\sigma_{i}^{2}=1^{T}V_{i}1,$$

and **1** is the unit vector **1**=(1,0,…,0)^T^. The univariate estimate is

$${\tilde{\beta }}_{1}=\frac{\sum \sigma_{i}^{-2}y_{i1}}{\sum \sigma_{i}^{-2}}$$

with variance

$$\text{var}\left({\tilde{\beta }}_{1}\right)=\frac{1}{\sum \sigma_{i}^{-2}}.$$

Under model (1), both ${\hat{\beta }}_{1}$ and ${\tilde{\beta }}_{1}$ are unbiased and normally distributed estimates of *β*_1_, and so to compare their statistical properties all we need to know is the efficiency *E*, which is defined by

(5) $$E=\frac{\text{var}({\hat{\beta }}_{1})}{\text{var}({\tilde{\beta }}_{1})}=\Omega_{11}\sum \sigma_{i}^{-2}=\frac{1}{n}{\overset{¯}{V}}_{11}\sum {\left(1^{T}V_{i}1\right)}^{-1}.$$

Necessarily, *E*⩽1 as the maximum likelihood estimate ${\hat{\beta }}_{1}$ is fully efficient. The smaller is *E*, the greater is the relative contribution of the secondary outcomes, suggesting 1−*E* as a measure of the role of the secondary outcomes in the multivariate estimate of the primary treatment effect. This combines the information in the secondary outcomes of all the studies in the meta‐analysis and so 1−*E* can be thought of as a measure of *total* borrowing of strength, which is equivalent to ${\text{BoS}}_{r}^{\mathrm{RV}}$ in the notation of Jackson *et al*. (2017) (section 2.2). However, the simpler notation 1−*E* emphasizes its dependence on a basic statistical concept which may open up useful interpretations taken from other areas of statistics: a possibility that is not immediately obvious from the earlier notation.

A simple example here is the familiar interpretation of efficiency in terms of sample size: for an inefficient estimate (efficiency *E*) to match the accuracy that a fully efficient estimate (efficiency 1) can achieve with a sample size of *n*, the sample size would have to be increased from *n* to *n*/*E*. Similarly, in meta‐analysis, the extra information which the secondary outcomes of *n* studies give to the estimation of *β*_1_ can be thought of as like the extra information that we would obtain in univariate meta‐analysis if we could measure the primary outcomes of a further *n*(1−*E*)/*E* studies. For example, if there are nine studies (*n*=9) and *E*=0.9, the advantage of using multivariate instead of univariate meta‐analysis is like finding the data for one more study. This simple idea will be used several times in the analysis of the hypertension example in Section 3.2 below.

The last expression in equation (5) is the ratio of the (1,1) element of the harmonic mean of the *V*_*i*_s to the harmonic mean of the (1,1) elements of the *V*_*i*_s. These are the same thing if the *V*_*i*_s are all the same, in which case *E*=1. If the *V*_*i*_s are different then *E*⩽1, which suggests another interpretation of 1−*E* as a measure of the variation of the matrices *V*_*i*_ about their harmonic average $\overset{¯}{V}$. This is analogous to the usual interpretation of the coefficient of variation (the ratio of standard deviation to the mean) as a simple relative measure of the variation of a univariate sample about its arithmetic mean.

These calculations are comparing the relative contributions which the primary and secondary outcomes make to the estimation of *β*_1_ by using all *n* studies in the meta‐analysis. To break this down into the contributions of individual studies, define, for any study with inverse variance matrix *V* ^−1^,

(6) $$T\left(V^{-1}\right)=\frac{{\left[\Omega V^{-1}\Omega \right]}_{11}}{\Omega_{11}}=\frac{1}{n}\frac{{\left[\overset{¯}{V}V^{-1}\overset{¯}{V}\right]}_{11}}{{\overset{¯}{V}}_{11}}.$$

We write the argument of equation (6) as *V* ^−1^ rather than *V* to reflect the fact that all of the formulae for multivariate meta‐analysis that were presented earlier involve the study variances *V*_*i*_ only through their inverses $V_{i}^{-1}$. As we shall see in Section 2.2, this also simplifies the notation in cases where there are missing data. Clearly, equation (6) is a function of two arguments, *V* ^−1^ and $\overset{¯}{V}$, and so equation (6) has further simplified the notation by suppressing the second argument. We can do this because we are mainly interested in the contributions of individual studies within the context of a *given* observed meta‐analysis, in which case we can treat $\overset{¯}{V}$ as if it was fixed.

We use the function *T*(*V* ^−1^) to investigate the role of individual studies in three different ways, analogous to the definitions of influence in regression analysis.

(a)*Direct interpretation*: from equation (3),
  $$\text{var}\left({\hat{\beta }}_{1}\right)=\Omega_{11}=\sum {\left[\Omega V_{i}^{-1}\Omega \right]}_{11}=\Omega_{11}\sum T\left(V_{i}^{-1}\right).$$
  Hence, $T(V_{i}^{-1})$ is the proportional contribution of the *i*th study to the variance of ${\hat{\beta }}_{1}$, proportional in the sense that
  $$\sum T(V_{i}^{-1})=1.$$
  In univariate meta‐analysis, $V_{i}^{-1}=\sigma_{i}^{-2}$ and equation (6) gives $T\left(V_{i}^{-1}\right)=\sigma_{i}^{-2}/\Sigma_{i}\sigma_{i}^{-2}$ which is just the weight of the *i*th study in the weighted average ${\hat{\beta }}_{1}$. When *p*⩾2, $T(V_{i}^{-1})$ can still be interpreted as the weight of the *i*th study in multivariate meta‐analysis, agreeing with the weight *w*_*ir*_ that is derived from an orthogonal decomposition of the score function in Jackson *et al*. (2017), section 3. However, the function *T*(*V* ^−1^) is not restricted to the *V*s which happen to be represented in the meta‐analysis.
(b)*Add‐one‐in interpretation*: if *n* is large and the variance matrix *V* is of the same order of magnitude as $\overset{¯}{V}$, then, under reasonable conditions on the matrices involved,
  (7) $${\left(I+n^{-1}\overset{¯}{V}V^{-1}\right)}^{-1}=I-n^{-1}\overset{¯}{V}V^{-1}+O\left(n^{-2}\right).$$
  Post‐multiplying each side of equation (7) by $n^{-1}\overset{¯}{V}$ and using equation (4), we obtain the approximation
  (8) $${\left[{\left(\sum_{j=1}^{n}V_{j}^{-1}+V^{-1}\right)}^{-1}\right]}_{11}=\Omega_{11}\left\{1-T\left(V^{-1}\right)\right\}+O\left(n^{-3}\right).$$
  The left‐hand side of equation (8) is the updated variance of ${\hat{\beta }}_{1}$ if we add a new study with inverse variance *V* ^−1^ to the meta‐analysis. So, for large *n*,* T*(*V* ^−1^) is the proportional *decrease* in $\text{var}({\hat{\beta }}_{1})$.
(c)*Leave‐one‐out interpretation*: replacing *V* by −*V*_*i*_ in equation (8) similarly shows that $T(V_{i}^{-1})$ is the proportional *increase* in $\text{var}({\hat{\beta }}_{1})$ if the *i*th study is removed from the meta‐analysis.

The first of these properties is exact, but the second and third are only asymptotic (large *n*) approximations. This reflects differences in the background studies being assumed for the add‐one‐in and hold‐one‐out calculations, i.e. differences in the second argument $\overset{¯}{V}$ in equation (6). For example, if a study we are thinking of adding in happens to have the same variance *V* as an existing study which we are thinking of leaving out, then the common value of *T*(*V* ^−1^) suggests that the two effects would be the same. But one is defining this study's contribution in terms of the difference between having *n*+1 studies and *n* studies, whereas the other is comparing *n*−1 with *n* studies. If *n* is large there is no material difference between the two. Essentially, the add‐one‐in and hold‐one‐out approximations are ignoring the effect that adding or subtracting studies has on the value of $\overset{¯}{V}$. These distinctions are analogous to the different definitions of residuals and influence in other areas of statistics. See Section 3 below for a clearer illustration of some of these points in the simpler context of bivariate meta‐analysis (*p*=2).

The definition of *E* in equation (5) arises from comparing $\text{var}({\hat{\beta }}_{1})$ with the value of this variance if only the primary outcomes had been measured across the whole of the meta‐analysis. Similarly, for investigating the role of individual studies, we can ask what happens to $\text{var}({\hat{\beta }}_{1})$ if we add in an extra study with variance matrix *V* but only take account of its primary outcome estimate *y*_1_∼*N*(*β*_1_,*σ* ^2^) with *σ* ^2^=**1** ^T^ *V* **1**. This will add *σ* ^−2^(*y*_1_−*β*_1_) to the score function (2) for the scalar *β*_1_ but will add nothing to the score function for the secondary outcomes. Hence the contribution to the vector score function for the estimation of the complete vector *β* is

$$V_{*}^{-1}\left(y-\beta \right),$$

where the matrix $V_{*}^{-1}$ is defined as

(9) $$V_{*}^{-1}=\sigma^{-2}11^{T}={\left(1^{T}V1\right)}^{-1}11^{T},$$

the *p*×*p* matrix with *σ* ^−2^ in the (1,1) position, and 0 everywhere else. The relative decrease in $\text{var}({\hat{\beta }}_{1})$ is therefore (approximately)

(10) $$T\left(V_{*}^{-1}\right)=T\left\{{\left(1^{T}V1\right)}^{-1}11^{T}\right\}.$$

We define the *borrowing of strengthB*(*V* ^−1^) of a study with variance matrix *V* to be the difference between equations (6) and (10):

(11) $$B\left(V^{-1}\right)=T\left(V^{-1}\right)-T\left\{{\left(1^{T}V1\right)}^{-1}11^{T}\right\}.$$

This measures the contribution that the secondary outcomes of this particular study makes to $\text{var}({\hat{\beta }}_{1})$ on top of the contribution that is made by its primary outcome. If *B*(*V* ^−1^) is 0 then nothing is gained by observing the secondary outcomes. The notation *T*(*V* ^−1^) refers to the total contribution of a study; the notation *B*(*V* ^−1^) refers to the borrowing of strength, i.e. how much of this proportional increase in precision is contributed by the secondary outcomes.

Although the formula for *T*(*V* ^−1^) is only an asymptotic approximation for the variance effect of adding a new study, as noted above we obtain exact results when adding over the existing studies. We can similarly add the univariate contributions (10) over the existing studies to give $\Omega_{11}^{-1}$ times

$${\left[\Omega \left(\sum \sigma_{i}^{-2}11^{T}\right)\Omega \right]}_{11}={\left[\left(\Omega 1\right){\left(\Omega 1\right)}^{T}\right]}_{11}\sum \sigma_{i}^{-2}={\left(\Omega_{11}\right)}^{2}\sum \sigma_{i}^{-2}.$$

It follows that

$$\sum T\left\{{\left(1^{T}V_{i}1\right)}^{-1}11^{T}\right\}=\Omega_{11}\sum \sigma_{i}^{-2}=E,$$

and so

(12) $$\sum B(V_{i}^{-1})=1-E.$$

This confirms that the efficiency of univariate meta‐analysis can be interpreted as the total of the proportional variance contributions of all the primary outcomes, and that the sum of the borrowing of strengths of these studies is the proportion of the total variance which is attributable to the secondary outcomes. For studies within the meta‐analysis, $B(V_{i}^{-1})$ is equivalent to ${\text{BoS}}_{\mathrm{ir}}^{\mathrm{SD}}$ in the notation of Jackson *et al*. (2017), section 2.4, and the additivity property (12) is implied by equations (11) and (12) of that section.

Both the functions *T*(*V* ^−1^) and *B*(*V* ^−1^) are linear functions in the sense that, for any positive scalar constant *k*,

(13) $$T\left(kV^{-1}\right)=kT\left(V^{-1}\right)$$

and

(14) $$B\left(kV^{-1}\right)=kB\left(V^{-1}\right).$$

Now multiplying the matrix *V* ^−1^ by *k* is like increasing the study sample size by the factor *k* while keeping the relative magnitudes of the elements of *V* ^−1^ the same. We can think of these relative magnitudes as determined by the design of the study—characteristics of the population from which we are sampling. The actual magnitudes of the elements of *V* ^−1^ are then determined by the sample size. Property (13) confirms that, if we add a new study to the meta‐analysis and double its sample size, then the decrease in variance will double. Property (14) shows that, if a study gives no borrowing of strength so that *B*(*V* ^−1^)=0, then *B*(*kV* ^−1^)=0 for all *k*. So whether or not a study offers any borrowing of strength depends only on the study's design and not on its sample size.

Riley (2009) noted that if the *V*_*i*_s are all the same then there is no borrowing of strength, and so the secondary outcomes are then irrelevant as far as estimating *β*_1_ is concerned. This follows immediately from the above formulation, since equation (6) would then give

(15) $$T\left({\overset{¯}{V}}^{-1}\right)=\frac{1}{n}=T\left\{{\left(1^{T}\overset{¯}{V}1\right)}^{-1}11^{T}\right\},$$

and hence $B({\overset{¯}{V}}^{-1})=0$. And so, if all the *V*_*i*_s are the same, $V_{i}=\overset{¯}{V}$ and so $B(V_{i}^{-1})=0$ for all *i*; hence *E*=1. This also follows from a simple argument of sufficiency: if $V_{i}=\overset{¯}{V}$ for all *i* then the score function (2) is exactly equivalent to that of a single study with $V=\overset{¯}{V}$ and *y*=Σ*y*_*i*_. But for any single study the estimate of *β*_*j*_ is simply the *j*th treatment effect estimate *y*_*j*_. As Riley (2009) implies, and already found here, borrowing of strength can arise only if there are differences between the *V*_*i*_s. More generally, for there to be any borrowing of strength, these differences must not be simply a matter of different sample sizes, but substantive differences in the background and research methods that are used in each study. This generalizes the special case of two groups of bivariate studies with proportional *V*_*i*_s that was discussed in Jackson *et al*. (2017), section 2.2.1.

When the *V*_*i*_s differ and *E*<1, as will usually be the case in practice, result (15) still holds for any study with $V=k\overset{¯}{V}$ for some scalar *k*, and so such a study will also give no borrowing of strength. We could describe such a study as one with ‘average design’. This suggests that it will tend to be the studies which are most atypical in terms of design which contribute most borrowing of strength. Studies whose designs are fairly typical of the meta‐analysis as a whole are likely to give little or no borrowing of strength, regardless of their sample sizes. Exactly what this means will be investigated further in Sections 3 and 4.

### 2.2 Missing outcomes

The univariate effect in equation (10) is for a study in which only the primary outcome is observed. More generally, suppose that only *q* of the *p* outcomes are observed; outcomes *y*_*j*_ with *j*=*j*_1_,*j*_2_,…,*j*_*q*_, with the remaining *p*−*q* outcomes assumed to be missing at random. We can think of this as selecting a *q*‐dimensional subvector from the *p*×1 vector *y*, which we can write as *J* ^T^ *y* where *J* is the *p*×*q* incidence matrix

$$J_{\mathrm{jk}}=\left\{\begin{array}{cc} 1 & \text{if}\;j=j_{k}\\ 0 & \text{otherwise} \end{array}\right.\text{for}\;j=1,2,\ldots ,p,\;k=1,2,\ldots ,q.$$

Matrix *J* is simply the matrix of 0s and 1s which picks out the required components—the first column has 1 in row *j*_1_, the second has 1 in row *j*_2_, and so on, with all other elements set to 0. Such a study's contribution to the score function for the corresponding subvector of *β* is then

(16) $${\left(J^{T}VJ\right)}^{-1}J^{T}\left(y-\beta \right).$$

There is no contribution to the score function for the missing outcomes, and so this study's contribution to the score function for the complete vector *β* is expression (16) padded out with 0s for each of the unobserved outcomes, namely

$$V_{*}^{-1}\left(y-\beta \right),$$

where now

(17) $$V_{*}^{-1}=J{\left(J^{T}VJ\right)}^{-1}J^{T}.$$

Thus, to fit the multivariate meta‐analysis model when one or more of the studies has missing outcome estimates, we simply use the complete‐data method as before but with the inverse of *V*_*i*_ for each incomplete studies replaced by the appropriate matrix (17).

If all outcomes are measured, then *q*=*p*,* J* is the *p*×*p* identity matrix and $V_{*}^{-1}=V^{-1}$ as expected. If only the primary outcome is measured, then *J*=1 and $V_{*}^{-1}$ is the previous case (9). Of particular interest is when only the secondary outcomes are measured, since in this case we have a study which cannot be included in a univariate analysis of the primary outcome but can be included in a multivariate analysis which can then allow information about the unobserved primary outcome to be imputed from the observed values of the secondary outcomes. In this case, *J* is the *p*×(*p*−1) matrix consisting of the (*p*−1)×(*p*−1) identity matrix supplemented with a row of 0s along the top.

Some care is needed in interpreting the notation $V_{*}^{-1}$. By replacing *V* ^−1^ with $V_{*}^{-1}$ for studies with missing outcomes, the usual formulae for maximum likelihood estimation that was set out earlier in this section continue to apply even if some, or even all, of the studies in the meta‐analysis have one or more missing outcomes. But, despite the notation, $V_{*}^{-1}$ cannot be interpreted as a matrix inverse (it is singular), or as the known value of *V* ^−1^ for an incomplete study. In reality, all the elements of *V* ^−1^ are unknown parameters, but with complete data we follow the usual convention of assuming that these are known because they can be consistently estimated from the within‐study data. However, with incomplete outcomes, only the submatrix *J* ^T^ *VJ* of the full matrix *V* is estimable, and so we have an estimate of $V_{*}^{-1}$ but not of *V* ^−1^. The rows and columns of 0s in $V_{*}^{-1}$ imply that various unidentifiable correlation parameters within *V* ^−1^ are being artificially set to 0. An equation such as equation (10) means that the contribution of an incomplete study to the variance of $\hat{\beta }$ is *as if* $V^{-1}=V_{*}^{-1}$. It does not mean that $V^{-1}=V_{*}^{-1}$ in the usual sense of a mathematical equality.

This discussion gives a formal justification for the more informal data augmentation view that was taken by Riley (2009) and Jackson *et al*. (2011), who referred to missing outcomes as equivalent to setting their variances to ∞ and their correlations to 0.

## 3 Borrowing of strength in bivariate meta‐analysis

### 3.1 The borrowing‐of‐strength plot

In bivariate meta‐analysis, with only one secondary outcome, we can obtain reasonably simple explicit expressions for all the quantities that were discussed in the previous section. In particular, the finding that borrowing of strength depends on differences between the *V*_*i*_s can be given a constructive interpretation in terms of residuals in a regression model.

In the bivariate case, suppose that the variance matrix *V*_*i*_ of *y*_*i*_=(*y*_*i*1_,*y*_*i*2_)^T^ is

$$V_{i}=\left(\begin{array}{cc} \sigma_{i}^{2} & \rho_{i}\sigma_{i}\nu_{i}\\ \rho_{i}\sigma_{i}\nu_{i} & \nu_{i}^{2} \end{array}\right)\;\;.$$

So (*σ*_*i*_,*ν*_*i*_) are the standard errors of (*y*_*i*1_,*y*_*i*2_), *ρ*_*i*_ is the correlation between them, and the inverse of *V*_*i*_ is

(18) $$V_{i}^{-1}=\frac{1}{1-\rho_{i}^{2}}\left(\begin{array}{cc} \sigma_{i}^{-2} & -\rho_{i}\sigma_{i}^{-1}\nu_{i}^{-1}\\ -\rho_{i}\sigma_{i}^{-1}\nu_{i}^{-1} & \nu_{i}^{-2} \end{array}\right)\;\;.$$

Adding equation (18) over the *n* studies, and taking the inverse, gives the harmonic mean

(19) $$\overset{¯}{V}=n\Omega =\frac{n}{s_{11}s_{22}-s_{12}^{2}}\left(\begin{array}{cc} s_{22} & s_{12}\\ s_{12} & s_{11} \end{array}\right)$$

where (*s*_11_,*s*_22_,*s*_12_) are weighted between‐studies sums of squares and products of the outcome accuracies $\left(\sigma_{i}^{-1},\nu_{i}^{-1}\right)$,

(20) $$s_{11}=\sum \frac{\sigma_{i}^{-2}}{1-\rho_{i}^{2}},$$

(21) $$s_{22}=\sum \frac{\nu_{i}^{-2}}{1-\rho_{i}^{2}},$$

(22) $$s_{12}=\sum \frac{\rho_{i}}{1-\rho_{i}^{2}}\sigma_{i}^{-1}\nu_{i}^{-1},$$

with weights depending on different functions of the within‐study correlations *ρ*_*i*_. As expected, each of these quantities retains the feature of a harmonic average.

Apart from these differences in the weights, equations (20), (21), (22) are like the second‐order absolute sample moments of the *n* pairs $\left(\sigma_{i}^{-1},\nu_{i}^{-1}\right)$, suggesting a through‐the‐origin linear regression model in which we can examine the extent to which a study's primary accuracy *σ* ^−1^ can be predicted from its secondary accuracy *ν* ^−1^. Allowing for the different weights, consider predicting *u*_*i*_ from *v*_*i*_, where

(23) $$\begin{array}{cc} u_{i} & =\frac{\rho_{i}}{{\left(1-\rho_{i}^{2}\right)}^{1/2}}\sigma_{i}^{-1},\\ v_{i} & =\frac{1}{{\left(1-\rho_{i}^{2}\right)}^{1/2}}\nu_{i}^{-1}. \end{array}$$

If we plot the *n* observed values of *u*_*i*_ against the corresponding values of *v*_*i*_, the least squares slope through the origin is

$$\frac{\sum u_{i}v_{i}}{\sum v_{i}^{2}}=\frac{s_{12}}{s_{22}},$$

and so the least squares prediction line is

(24) $$\hat{u}=\frac{s_{12}}{s_{22}}v=\frac{s_{12}}{{\left(1-\rho^{2}\right)}^{1/2}s_{22}}\nu^{-1}.$$

Requiring the regression line to go though the origin is a natural requirement, since if we know that a study has a very small sample size then we know in advance that both *u* and *v* will be close to 0. The definitions of *u* and *v* in expression (23) have assumed complete data, but studies with missing data can also be included as in Section 2.2. If only the primary outcome estimate in the *i*th study is observed, then we take both $\nu_{i}^{-1}$ and *ρ*_*i*_ to be 0, and so *u*_*i*_=*v*_*i*_=0. If only the secondary outcome estimate is observed, we take $\sigma_{i}^{-1}$ and *ρ*_*i*_ to be 0, leading to *u*_*i*_=0 and $v_{i}=\nu_{i}^{-1}$.

The plot of the *n* values of *u*_*i*_ against their predicted values ${\hat{u}}_{i}$ turns out to be closely related to the borrowing‐of‐strength function $B(V_{i}^{-1})$ that was defined in Section 2. Using equations (18) and (19), and evaluating the required matrix terms explicitly, we obtain

$$T\left(V_{i}^{-1}\right)=\frac{{\left[\Omega V_{i}^{-1}\Omega \right]}_{11}}{\Omega_{11}}=\frac{s_{22}^{2}\sigma_{i}^{-2}-2s_{12}s_{22}\rho_{i}\sigma_{i}^{-1}\nu_{i}^{-1}+s_{12}^{2}\nu_{i}^{-2}}{s_{22}\left(s_{11}s_{22}-s_{12}^{2}\right)\left(1-\rho_{i}^{2}\right)}.$$

Rewriting *ν*_*i*_ and *σ*_*i*_ in terms of *u*_*i*_ and *v*_*i*_, and completing the square, gives

$$T\left(V_{i}^{-1}\right)=\Omega_{11}\;\left\{\frac{1-\rho_{i}^{2}}{\rho_{i}^{2}}u_{i}^{2}+{\left(u_{i}-\frac{s_{12}}{s_{22}}v_{i}\right)}^{2}\right\}\;\;.$$

The first term in the outer brackets is just $\sigma_{i}^{-2}$, which is proportional to the univariate variance contribution of the primary outcome, and so the borrowing of strength is just the second term

(25) $$B\left(V_{i}^{-1}\right)=\Omega_{11}\;{\left(u_{i}-\frac{s_{12}}{s_{22}}v_{i}\right)}^{2}=\Omega_{11}{\left(u_{i}-{\hat{u}}_{i}\right)}^{2}.$$

Thus $B(V_{i}^{-1})$ is proportional to the squared residual of the point $\left({\hat{u}}_{i},u_{i}\right)$ from the diagonal prediction line $u=\hat{u}$. For any other study with inverse variance *V* ^−1^, *B*(*V* ^−1^) is similarly proportional to the squared residual of its point $\left(\hat{u},u\right)$ from the line and so indicates the (approximate) decrease in $\text{var}({\hat{\beta }}_{1})$ which we would obtain if we were to add this study to the meta‐analysis. The proportionality factor is

$$\Omega_{11}=\text{var}\left({\hat{\beta }}_{1}\right)=\frac{s_{22}}{s_{11}s_{22}-s_{12}^{2}}.$$

If the *i*th study has missing data, $\left({\hat{u}}_{i},u_{i}\right)$ is either (0,0) when the secondary outcome estimate is missing, or $\left(\left(s_{12}/s_{22}\right)\nu_{i}^{-1}),0\right)$ when the primary outcome estimate is missing. In the first case, the point is always on the line and so, as expected, there can be no borrowing of strength. In the second case, the point is down on the horizontal axis and so will generally have a non‐zero residual and so, again as expected, will contribute at least some borrowing of strength.

The $\left({\hat{u}}_{i},u_{i}\right)$ plot is easier to interpret if we first scale *u*_*i*_ and ${\hat{u}}_{i}$ by the factor $\Omega_{11}^{1/2}$, giving

$$w_{i}=\Omega_{11}^{1/2}u_{i}={\left(\frac{s_{22}}{s_{11}s_{22}-s_{12}^{2}}\right)}^{1/2}\frac{\rho_{i}\sigma_{i}^{-1}}{{\left(1-\rho_{i}^{2}\right)}^{1/2}}$$

and

$${\hat{w}}_{i}=\Omega_{11}^{1/2}{\hat{u}}_{i}={\left\{\frac{s_{12}^{2}}{s_{22}\left(s_{11}s_{22}-s_{12}^{2}\right)}\right\}}^{1/2}\frac{\nu_{i}^{-1}}{{\left(1-\rho_{i}^{2}\right)}^{1/2}}.$$

We call the scatter plot of *w*_*i*_ against ${\hat{w}}_{i}$ the *borrowing‐of‐strength plot*. Now the *i*th squared residual from the diagonal line, ${\left(w_{i}-{\hat{w}}_{i}\right)}^{2}$, is *equal* to $B(V_{i}^{-1})$. The combined variance contributions of the secondary outcomes in the meta‐analysis are indicated by the scatter of the points about the diagonal regression line. If the points all lie on the line then $B(V_{i}^{-1})=0$ for all *i* and so *E*=1. More generally, we can show from the earlier formulae that

(26) $$1-E=\sum {\left(w_{i}-{\hat{w}}_{i}\right)}^{2},$$

and so 1−*E* is equal to the residual sum of squares of the points in the borrowing‐of‐strength plot.

To aid interpretation of the borrowing‐of‐strength plot, equation (26) means that, for efficiency *E*, the root‐mean‐squared distance of the points from the diagonal line $w=\hat{w}$ is

$$\overset{¯}{d}={\left(\frac{1-E}{n}\right)}^{1/2}.$$

For example, to achieve 90% efficiency, the root‐mean‐squared distance is $\overset{¯}{d}={\left(10n\right)}^{-1/2}$. This is indicated on the borrowing‐of‐strength plot by the two parallel lines

(27) $$w=\hat{w}\pm {\left(\frac{1}{10n}\right)}^{1/2}.$$

These lines give a visual benchmark for interpreting residuals in terms of efficiency. If the points are predominantly inside, or predominantly outside, these lines, then the efficiency of univariate meta‐analysis is likely to be greater than, or less than, 0.9. As noted previously in Section 2.1, an efficiency of 90% indicates that the information that is gained from the secondary outcomes in multivariate meta‐analysis is like the extra information which would be available in univariate meta‐analysis if we had an additional *n*/9 studies.

Equation (25) also gives us the necessary and sufficient condition for a study to give no borrowing of strength. If $\left({\hat{u}}_{i},u_{i}\right)$ lies on the line, then *u*_*i*_=(*s*_12_/*s*_22_)*v*_*i*_ and so

(28) $$\frac{\rho_{i}\sigma_{i}\nu_{i}}{\sigma_{i}^{2}}=\frac{s_{12}}{s_{22}}.$$

The left‐hand side of equation (28) is the ratio of the covariance element in *V*_*i*_ (*V*_*i*12_) to its primary diagonal element *V*_*i*11_, whereas the right‐hand side is the ratio of the corresponding elements of Ω, or of $\overset{¯}{V}$. For no borrowing of strength these are equal, and so

(29) $$\frac{V_{i12}}{V_{i11}}=\frac{{\overset{¯}{V}}_{12}}{{\overset{¯}{V}}_{11}}.$$

Previously we noted that a study with $V_{i}=k\overset{¯}{V}$ for some scalar constant *k* gives no borrowing of strength. This is a sufficient but not necessary condition—all we need is that the top row (or left‐hand column) of *V*_*i*_ is proportional to the top row (or left‐hand column) of $\overset{¯}{V}$. In particular, there is no requirement on the secondary variance $\nu_{i}^{2}$ *per se*. We show in Section 4 that this generalizes to any number of secondary outcomes.

The borrowing‐of‐strength plot also illustrates two other aspects of borrowing of strength which were discussed in Section 2. Firstly, for studies in the meta‐analysis, $B(V_{i}^{-1})$ is the proportional contribution of the *i*th secondary outcome estimate to $\text{var}({\hat{\beta }}_{1})$ (the direct interpretation), but, for a study outside the meta‐analysis, *B*(*V* ^−1^) is only the approximate (large *n*) contribution which the secondary outcome estimate would make if this study were added to the meta‐analysis (the add‐one‐in interpretation). We see the nature of this approximation in the borrowing‐of‐strength plot. The line $w=\hat{w}$ is the least squares line of best fit (through the origin) for the *n* points $\left({\hat{w}}_{i},w_{i}\right)$. But, if we add in the new study, the value of the scale factor $\Omega_{11}^{1/2}$ will change, affecting the co‐ordinates for all the studies. So the residual of the new point from the line fitted by least squares to the enhanced data will not be the same as the residual from the line calculated from the original *n* studies alone. If *n* is large then adding one more study will have only a small effect on the fitted line, and so these two residuals will be similar.

Secondly, we have noted the linear property of the function *B*(*V* ^−1^) in equation (14). If we multiply *V* ^−1^ by *k* then both *w* and $\hat{w}$ are scaled by the factor √*k* and so the squared residual from the diagonal line is scaled by the original factor *k*, which means that *B*(*kV* ^−1^)=*kB*(*V* ^−1^) as required. If *V* ^−1^ gives no borrowing of strength then the point will simply move up or down the diagonal line according to the value of *k*.

### 3.2 Example

**Figure 1:**
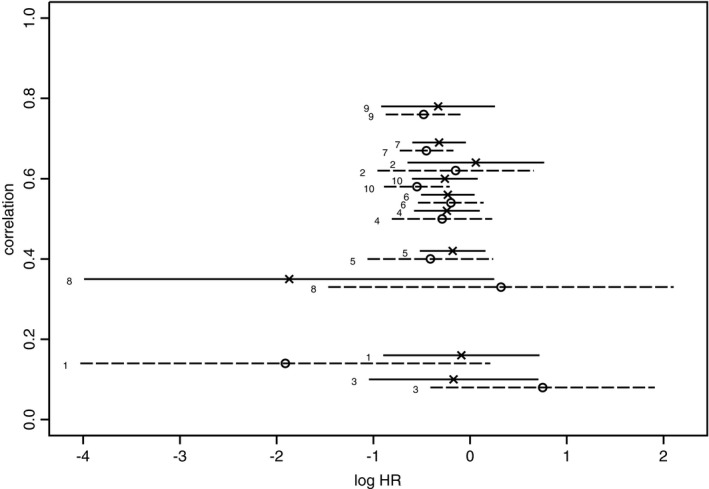
Graph illustrating the raw data for the example (the plotted points and horizontal line segments show the within‐study estimates and 95% confidence intervals for the hazard ratios for CVD and for stroke within each of the 10 trials, plotted against within‐study correlation): ×, CVD estimate; 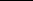, CVD confidence interval; ∘, stroke estimate; — —, stroke confidence interval

Fig. 1 illustrates data from 10 clinical trials designed to test the effectiveness of hypertension treatments in reducing the risk of subsequent diagnoses of cardio‐vascular disease (CVD) and stroke. This meta‐analysis was originally published by Wang *et al*. (2005) and discussed further in Riley *et al*. (2015) and Jackson *et al*. (2017). Each randomized controlled trial was well balanced between active treatment and placebo but varied widely in size, from under 200 patients in trial 3 to almost 7000 patients in trial 5 (the trial numbers are consistent with previous tables, e.g. Table 1 of Riley *et al*. (2015)). Fig. 1 shows individual trial data for two outcomes: the estimated log‐hazard‐ratio log(HR) for CVD, *y*_1_, and the estimated log(HR) for stroke, *y*_2_. Values of *y*_1_ (crosses) and *y*_2_ (circles) are plotted against the within‐study correlations *ρ*_*i*_, with the corresponding pairs of within‐study confidence intervals for *β*_1_ and *β*_2_ shown as the full and broken line segments respectively. The small numbers to the left of the confidence intervals identify the study numbers 1–10. The vertical co‐ordinates of some of the data in Fig. 1 have been slightly adjusted to aid clarity of the plot. Separate homogeneity tests of the values of *y*_1_ and *y*_2_ are both well consistent with fixed effects models, leading to univariate combined confidence intervals of (−0.374,−0.115) for CVD log(HR), and (−0.531,−0.235) for stroke log(HR). It is not at all obvious from Fig. 1 whether a bivariate approach, taking both outcomes into account, will lead to more accurate estimates and if so by how much.

**Table 1:** Sample sizes, values of (*σ*,*ν*,*ρ*), and percentage values of $T(V_{i}^{-1})$ and $B(V_{i}^{-1})$ for estimating *β*_1_ in the example

*Study*	*Sample size*	*σ*	*ν*	*ρ*	100*T*(*V* ^−1^)	100*B*(*V* ^−1^)
					(%)	(%)
1	1530	0.41	1.08	0.16	2.5	0.02
2	349	0.36	0.41	0.64	3.3	0.02
3	172	0.45	0.59	0.10	2.5	0.34
4	4798	0.17	0.26	0.52	14.4	0.15
5	6991	0.17	0.33	0.42	14.3	0.10
6	2651	0.14	0.17	0.62	21.6	0.23
7	4736	0.14	0.14	0.69	21.4	0.04
8	268	1.08	0.91	0.35	0.4	0.08
9	2391	0.30	0.20	0.78	5.3	0.53
10	4695	0.17	0.17	0.62	14.3	0.04
Total	28581				100.0	1.55

If the log‐hazard‐ratio for CVD is taken as the primary outcome, *y*_1_, the formulae in Section 2 give the respective univariate and multivariate estimates of *β*_1_ and their variances as

(30) $$\left.\begin{array}{cc} & {\tilde{\beta }}_{1}=-0.244,\\ & \text{var}({\tilde{\beta }}_{1})=0.00434,\\ & {\hat{\beta }}_{1}=-0.244,\\ & \text{var}({\hat{\beta }}_{1})=0.00427. \end{array}\right\}$$

**Figure 2:**
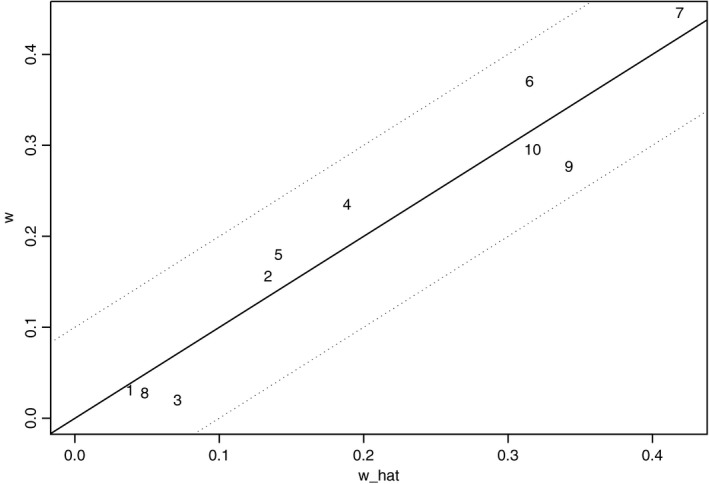
Borrowing‐of‐strength plot for estimating *β*_1_ (*E*=0.984)

The estimates are virtually identical. The ratio of the variances is the efficiency *E*=0.984, showing that in this example the stroke data give very little extra information for the assessment of CVD risk reduction. The last two columns of Table 1 give the total variance contribution $T(V_{i}^{-1})$ and the borrowing of strength $B(V_{i}^{-1})$ for each of the 10 studies, confirming that none of these studies gives any worthwhile contribution from the secondary outcome. We can check directly that the borrowing‐of‐strength figures add up to 1−*E*. Fig. 2 shows the corresponding borrowing‐of‐strength plot. Again we can check the theory by showing that the least squares slope of these points is 1, and that the residual sum of squares is 1−*E*=0.016. The two dotted lines are the 90% efficiency bars (27). All the points are well within these limits, confirming the high efficiency of univariate meta‐analysis and the minimal contribution of the secondary outcomes in this case.

**Figure 3:**
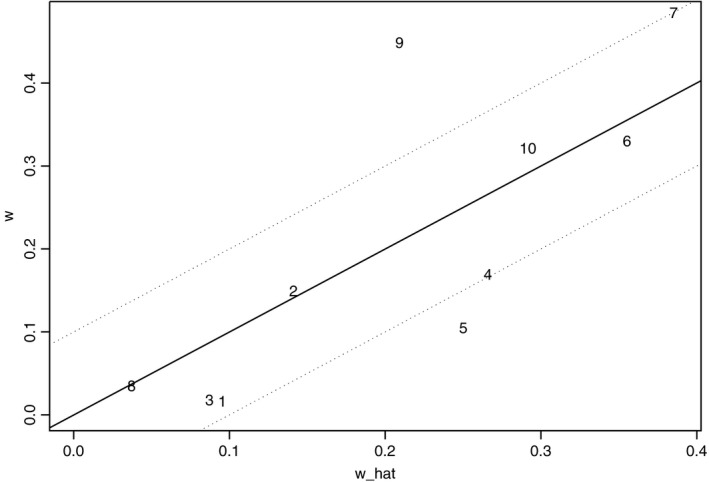
Borrowing‐of‐strength plot for estimating *β*_2_ (*E*=0.888)

**Figure 4:**
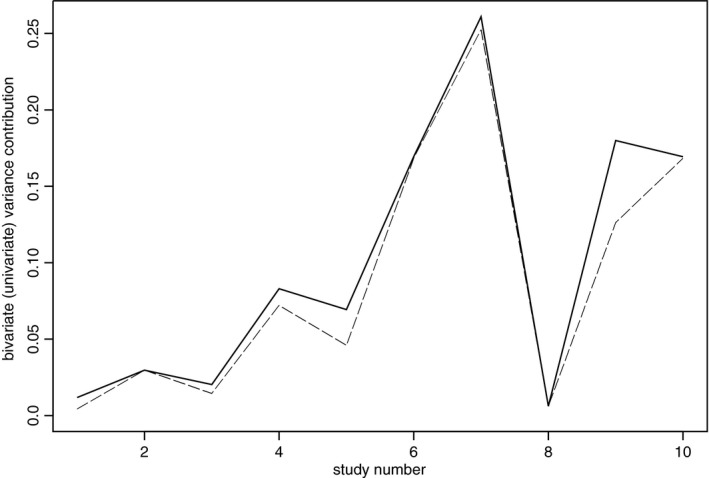
Study values of *T* (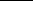, multivariate variance contribution) and *T*−*B* (— —, univariate variance contribution) for estimating *β*_2_ (*E*=0.888)

If the primary interest is to estimate log(HR) for stroke instead of CVD, then we use exactly the same formulae but with the notation reversed appropriately, retaining the same values of *ρ*_*i*_ and *s*_12_ but interchanging *y*_*i*1_ with *y*_*i*2_, *σ*_*i*_ with *ν*_*i*_ and *s*_11_ with *s*_22_. In terms of the original notation we are now estimating *β*_2_, giving

$$\begin{array}{cc} & {\tilde{\beta }}_{2}=-0.383,\\ & \text{var}({\tilde{\beta }}_{2})=0.00569,\\ & {\hat{\beta }}_{2}=-0.381,\\ & \text{var}({\hat{\beta }}_{2})=0.00505, \end{array}$$

with the new efficiency *E*=0.888. The two estimates are again very similar, but the multivariate method is now noticeably more accurate. The borrowing‐of‐strength plot for estimating *β*_2_ is shown in Fig. 3, which now shows a much greater dispersion about the regression line than in Fig. 2 (the mean‐squared spread of the residuals is now close to the dotted 90% efficiency lines). Fig. 4 illustrates the proportional contributions which the studies make to $\text{var}({\hat{\beta }}_{2})$. This is a line plot: the upper (full) line highlighting the values of $T(V_{i}^{-1})$ (total contributions); the lower (broken) line highlighting the corresponding values of $T\left(V_{i}^{-1}\right)-B\left(V_{i}^{-1}\right)$ (univariate contributions). The distance between the two lines matches the squared residuals in Fig. 3. The largest borrowing of strength comes from the ninth study, where the secondary outcome accounts for almost a third of the total variance contribution of that study. This study accounts for about a half of the total borrowing of strength of all the studies, although its sample size is by no means the largest (although it does have the largest correlation). The efficiency of 89% shows that the variance of the multivariate estimate of *β*_2_ is about 10% lower than the variance of the univariate estimate, which is roughly what we might expect if we could increase the size of a univariate meta‐analysis from 10 to 11 studies. In this sense, the value of including data on the 10 secondary outcomes can be likened to the value of having the primary outcome estimate of one additional study.

Comparing these two efficiencies shows that there is no symmetry in borrowing of strength: the values of *y*_1_ make a modest contribution to the accuracy of ${\hat{\beta }}_{2}$ but the values of *y*_2_ make almost no contribution to the accuracy of ${\hat{\beta }}_{1}$. More generally, we can show that, if *E*=1 (no borrowing of strength) when estimating *β*_1_, then *E* will be strictly less than 1 (positive borrowing of strength) for estimating *β*_2_ except in the special case of all the studies having the same correlation (as in Section 3.3).

There are no missing data in these trials. To illustrate the effect that missing outcomes might have had on this analysis, and to demonstrate the use of multivariate meta‐analysis when there are missing data, imagine that we wish to estimate the CVD risk *β*_1_ when both outcomes are available in trials 1–5 but only the stroke outcome is measured in the remaining trials 6–10. Then we obtain

$$\begin{array}{cc} & {\tilde{\beta }}_{1}=-0.175,\\ & \text{var}({\tilde{\beta }}_{1})=0.0117,\\ & {\hat{\beta }}_{1}=-0.196,\\ & \text{var}({\hat{\beta }}_{1})=0.00956. \end{array}$$

Inevitably, the variance of ${\hat{\beta }}_{1}$ is now considerably larger than the complete‐data case in equation (30). The efficiency of *E*=0.815 now reflects the difference between univariate meta‐analysis using only the first five trials and multivariate meta‐analysis using the information in all 10 trials. This value of *E* is roughly 5/6, which is the improvement in variance that we might expect to obtain if we could use univariate meta‐analysis with the number of trials increased from 5 to 6. In this sense, the value of including the five trials with missing primary outcomes can be likened to the value of having one further trial with complete data.

**Figure 5:**
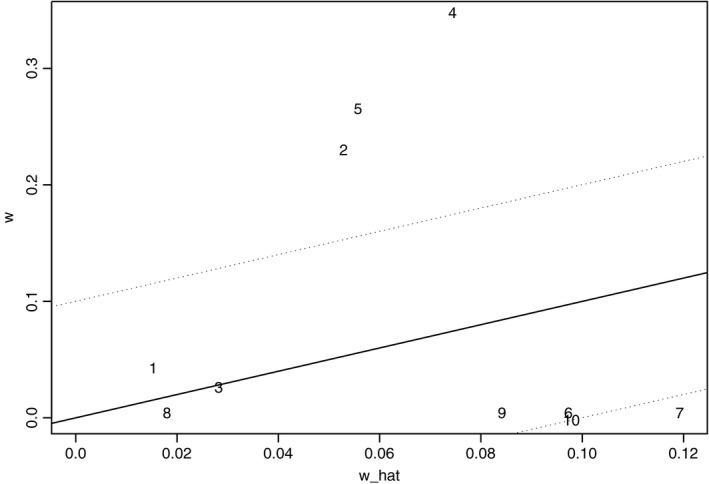
Borrowing‐of‐strength plot for estimating *β*_1_ with missing outcomes (*E*=0.815)

**Figure 6:**
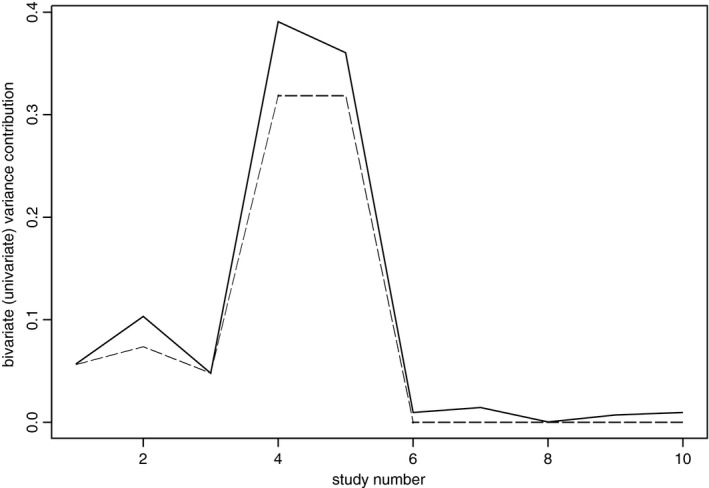
Study values of *T* (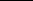, multivariate variance contribution) and *T*−*B* (— —, univariate variance contribution) for estimating *β*_1_ with missing outcomes (*E*=0.815)

Fig. 5 is the borrowing‐of‐strength plot for this missing data example. The points $\left({\hat{w}}_{i},w_{i}\right)$ for studies 1–5 are the same as in Fig. 2 except for a rescaling of the axes, but the five points for the missing studies are all moved vertically down to the horizontal axis. This completely alters the size of the residuals and hence the borrowing‐of‐strength figures for all the trials. Fig. 2 showed that, for estimating *β*_1_ with complete data, none of the 10 secondary values *y*_2_ makes any useful contribution on top of the contribution of the corresponding observed values of *y*_1_. So we might expect that with these missing data all of the borrowing of strength would come from trials 5–10 since in these trials *y*_1_ is no longer available. But this is not so, as shown in the variance contributions plot in Fig. 6 (using the same format as Fig. 4). Now we obtain

(31) $$\begin{array}{cc} & \sum_{1}^{5}B\left(V_{i}^{-1}\right)=0.144,\\ & \sum_{6}^{10}B\left(V_{*i}^{-1}\right)=0.041, \end{array}$$

where $V_{*i}^{-1}$ is the proxy matrix (9) for the *i*th trial: the 2×2 matrix with $\nu_{i}^{-2}$ as the lower diagonal element and 0s elsewhere. The sum of these two numbers in expression (31) is 0.185=1−*E* as expected, but the missing studies contribute only 22% of the total borrowing of strength. This illustrates one of the main points in Section 2.1, that the borrowing of strength that is given by a particular study depends on how typical that study is of the meta‐analysis as a whole, and only indirectly on the statistical characteristics of the study itself. Changing the later studies leaves studies 1–5 exactly the same but can drastically alter their borrowing of strength. We can also see a difference if we look at the estimation of *β*_2_ with the same pattern of missing data. We are again leaving trials 1–5 as before, but now trials 5–10 measure only the primary outcome. Now the borrowing of strengths $B(V_{i}^{-1})$ for the first five trials adds up to about 1%, which is less than the sum over the same trials in the complete‐data case of about 5% (Fig. 4).

### 3.3 The special case of equal within‐study correlations

A statistical understanding of the plotting co‐ordinates $\left({\hat{w}}_{i},w_{i}\right)$ in the borrowing‐of‐strength plot is complicated by the fact that the weighted sums of squares and products in equations (20), (21), (22) use different weights, which is also reflected in the different factors appearing in *u*_*i*_ and *v*_*i*_ in equation (23). However, if the *ρ*_*i*_s are constant, *ρ*_*i*_=*ρ*_0_ say, these differences in the weights can be absorbed into an overall scale factor, leading to a more transparent version of many of the formulae in Section 3.1. This special case is also of interest in its own right since, as will be discussed in Section 5, fitting the bivariate model with constant correlations can provide a useful sensitivity analysis in cases where the within‐study correlations are not provided by the study reports (Jackson *et al*., 2011).

Let $\left(s_{11}^{*},s_{22}^{*},s_{12}^{*}\right)$ be the ordinary (unweighted) sums of squares and products of the *n* accuracy pairs $\left(\sigma_{i}^{-1},\nu_{i}^{-1}\right)$. Then, if we imagine a scatter plot of $\sigma_{i}^{-1}$ against $\nu_{i}^{-1}$, the least squares line of best fit through the origin has slope $s_{12}^{*}/s_{22}^{*}$. Thus, for any given value of *ν* ^−1^, the least squares prediction of *σ* ^−1^ is

$$\hat{\sigma^{-1}}=\frac{s_{12}^{*}}{s_{22}^{*}}\nu^{-1}.$$

Relating this to the earlier notation gives

$$\left(u_{i},{\hat{u}}_{i}\right)=\frac{\rho_{0}}{{\left(1-\rho_{0}^{2}\right)}^{1/2}}\left(\sigma_{i}^{-1},\hat{\sigma_{i}^{-1}}\right),$$

and so the *i*th residual in the borrowing‐of‐strength plot is

$$w_{i}-{\hat{w}}_{i}={\left(\frac{\rho_{0}^{2}s_{22}^{*}}{s_{11}^{*}s_{22}^{*}-\rho_{0}^{2}s_{12}^{*2}}\right)}^{1/2}\left(\sigma_{i}^{-1}-\hat{\sigma_{i}^{-1}}\right).$$

So, with this slightly different scale factor, we can think of the borrowing‐of‐strength plot as little more than a linear regression of the within‐study accuracies of the primary outcomes plotted against the corresponding accuracies of the secondary outcomes. The fact that borrowing of strength is given by the least squares residuals again confirms that borrowing of strength is all a matter of how the variances of individual studies fit in with the overall pattern of variances in the meta‐analysis as a whole.

### 3.4 Borrowing of strength as a within‐study ratio

We have measured borrowing of strength in terms of *B*(*V* ^−1^): the variance contribution of a study's secondary outcome relative to the overall variance Ω_11_. We could instead consider the ratio *R*(*V* ^−1^)=*B*(*V* ^−1^)/*T*(*V* ^−1^): the contribution of the study's secondary outcome as a proportion of that study's total contribution to $\text{var}({\hat{\beta }}_{1})$. This removes the effect of any scale factor in *V*, so for a given meta‐analysis *R*(*V* ^−1^) is a function of just two quantities: *ρ*, the correlation between the outcomes, and *z*, the ratio of the standard errors,

$$z=\frac{\sigma }{\nu }.$$

The earlier formulae now give

(32) $$\begin{array}{cc} R(V^{-1}) & =\frac{\rho^{2}{\left(u-s_{22}^{-1}s_{12}v\right)}^{2}}{\rho^{2}{\left(u-s_{22}^{-1}s_{12}v\right)}^{2}+\left(1-\rho^{2}\right)u^{2}}\\ & =\frac{{\left(\rho -s_{22}^{-1}s_{12}z\right)}^{2}}{{\left(\rho -s_{22}^{-1}s_{12}z\right)}^{2}+1-\rho^{2}}. \end{array}$$

A contour plot of equation (32) against *ρ* and *z* gives a complete picture of how, within a given meta‐analysis (i.e. for a given value of the slope parameter *s*_12_/*s*_22_) a study's borrowing of strength, defined in this way, depends on individual study characteristics.

**Figure 7:**
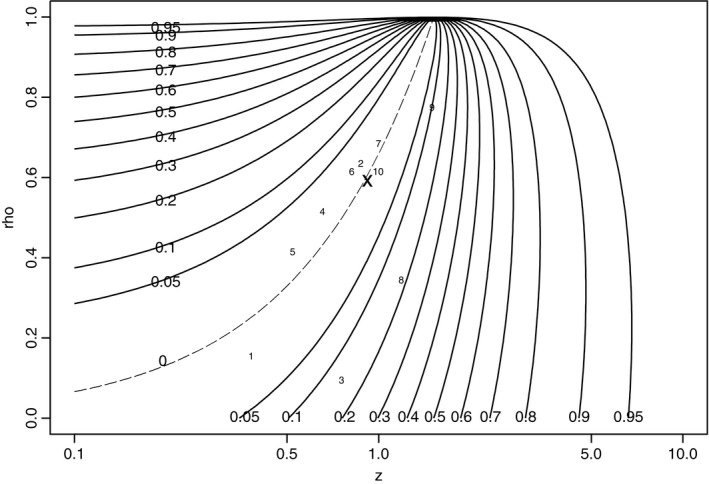
Contour plot of *R*(*V* ^−1^) for estimating *β*_1_ (*E*=0.984): the points labelled 1–10 show the values of (*z*,*ρ*) for the studies in the example; the point **X** indicates the corresponding point for matrix $\overset{¯}{V}$

Fig. 7 shows a contour plot of *R*(*V* ^−1^) for *s*_12_/*s*_22_=0.660: the value of the slope parameter found in the example in Section 3.2. Values of *ρ* are shown on the vertical axis; values of *z* are shown by using a log‐scale on the horizontal axis. The contour values are labelled along the bottom and up the left‐hand side of the plot. The broken line is the zero contour when *ρ*=0.660*z*: at these values there is no borrowing of strength. The contour plot shows that *R* is large when either *z* is large (*y*_1_ less accurate than *y*_2_), or when *z* is small (*y*_1_ more accurate than *y*_2_) *and ρ* is large (outcomes highly correlated). The smaller plotting symbols 1–10 in Fig. 7 show the values of (*z*,*ρ*) for the 10 studies in the example. Most of the points are fairly close to the zero contour: for only three of these studies is *R*(*V* ^−1^)>0.1, suggesting that *E* is close to 1, as found earlier. The plotting symbol **X** indicates the point (*z*,*ρ*) for a study with $V=\overset{¯}{V}$ defined in equation (4). This point corresponds to the harmonic mean of the 10 points labelled 1–10, and, as expected, lies on the zero contour (no borrowing of strength). The interpretation of a study's borrowing of strength as a contrast between *V* and $\overset{¯}{V}$ can be seen on the graph as the distance between the study's (*z*,*ρ*) point and the harmonic mean point **X**, measured in the direction that is orthogonal to the contours in that region.

If one of the outcomes is missing, *R*(*V* ^−1^) becomes $R(V_{*}^{-1})$ and so the point (*z*,*ρ*) lies on the horizontal axis, to the extreme left if *y*_2_ is missing and to the extreme right if *y*_1_ is missing, giving $R(V_{*}^{-1})$ equal to 0 and 1 respectively, as expected.

## 4 Borrowing of strength in multivariate meta‐analysis

### 4.1 Decomposing the variance contribution of an individual study

This section looks at the generalization of Section 3 to the multivariate case with *p*>2. Now the outcome estimates of the *i*th study are *y*_*i*_=(*y*_*i*1_,*y*_*i*2_) with *y*_*i*2_, the secondary outcome estimates, a (*p*−1)×1 vector. When *p*=2, all the formulae in this section reduce to the corresponding expressions that have already been seen in Section 3.

In the multivariate case, we write *V*_*i*_=var(*y*_*i*_) as the partitioned matrix

(33) $$V_{i}=\left(\begin{array}{cc} \sigma_{i}^{2} & \sigma_{i}\rho_{i}^{T}\Lambda_{i}\\ \sigma_{i}\Lambda_{i}\rho_{i} & \Lambda_{i}P_{i}\Lambda_{i} \end{array}\right)\;\;,$$

where $\sigma_{i}^{2}$ is the variance of *y*_*i*1_ as before, *ρ*_*i*_ is the (*p*−1)×1 vector of correlation coefficients between *y*_*i*1_ and *y*_*i*2_, *P*_*i*_ is the (*p*−1)×(*p*−1) correlation matrix of *y*_*i*2_ and Λ_*i*_ is the (*p*−1)×(*p*−1) diagonal matrix of the standard deviations of the components of *y*_*i*2_.

To simplify the algebra for calculating matrix inverses, define

(34) $$\left.\begin{array}{cc} & a_{i}=\rho_{i}^{T}P_{i}^{-1}\rho_{i},\\ & b_{i}=P_{i}^{-1}\rho_{i},\\ & C_{i}=P_{i}^{-1}+\frac{b_{i}b_{i}^{T}}{1-a_{i}}. \end{array}\right\}$$

Then, using a standard formula for the inverse of a partitioned matrix,

(35) $$V_{i}^{-1}=\frac{1}{1-a_{i}}\left(\begin{array}{cc} \sigma_{i}^{-2} & -\sigma_{i}^{-1}b_{i}^{T}\Lambda_{i}^{-1}\\ -\sigma_{i}^{-1}\Lambda_{i}^{-1}b_{i} & \left(1-a_{i}\right)\Lambda_{i}^{-1}C_{i}\Lambda_{i}^{-1} \end{array}\right)\;\;.$$

Some of this notation can be interpreted in terms of a multiple regression of the primary on the secondary outcome estimates within the *i*th study. The vector in the off‐diagonal partition of equation (35) is proportional to the vector of regression coefficients, and $\sigma_{i}^{2}\left(1-a_{i}\right)$ is the residual mean square. Thus *a*_*i*_ can be interpreted as the multiple correlation *R* ^2^ of this regression: *a*_*i*_=0 means that the primary and secondary outcome estimates are independent; *a*_*i*_=1 means that they are exactly linearly related.

Adding equation (35) over the *n* studies gives

(36) $$\Omega^{-1}=\sum V_{i}^{-1}=n{\left(\overset{¯}{V}\right)}^{-1}=\left(\begin{array}{cc} s_{11} & -s_{12}^{T}\\ -s_{12} & S_{22} \end{array}\right)\;\;,$$

where

$$\begin{array}{cc} & s_{11}=\sum \frac{\sigma_{i}^{-2}}{1-a_{i}},\\ & S_{22}=\sum \Lambda_{i}^{-1}C_{i}\Lambda_{i}^{-1},\\ & s_{12}=\sum \frac{\sigma_{i}^{-1}\Lambda_{i}^{-1}b_{i}}{1-a_{i}}. \end{array}$$

Thus the inverse of equation (36) is

(37) $$\Omega =\frac{\overset{¯}{V}}{n}=\frac{1}{s_{11}-s_{12}^{T}S_{22}^{-1}s_{12}}(\begin{array}{cc} 1 & s_{12}^{T}S_{22}^{-1}\\ S_{22}^{-1}s_{12} & \left(s_{11}-s_{12}^{T}S_{22}^{-1}s_{12}\right)S_{22}^{-1}+S_{22}^{-1}s_{12}s_{12}^{T}S_{22}^{-1} \end{array})$$

and so $\text{var}({\hat{\beta }}_{1})$ is

(38) $$\Omega_{11}=\frac{1}{s_{11}-s_{12}^{T}S_{22}^{-1}s_{12}}.$$

As before, the components of Ω^−1^ in equation (36) are weighted sums of squares and products of the precisions of the components of *y*_*i*_: $\sigma_{i}^{-1}$ for the primary outcome and the diagonal elements of $\Lambda_{i}^{-1}$ for the secondary outcomes. The scalar *s*_11_ is the same as in the bivariate case, *S*_22_ is the (*p*−1)×(*p*−1) matrix of weighted sums of squares and products for the secondary outcome precisions and *s*_12_ is the corresponding (*p*−1)×1 vector of weighted sums of cross‐products between the primary and secondary precisions. When *p*=2 these formulae reduce to the corresponding quantities in Section 3, with the matrix *S*_22_ becoming the scalar *s*_22_. In the bivariate case, the weights that are involved in these sums are also the same, since when *p*=2 the quantities that are defined in expression (34) reduce to the scalars

(39) $$\left.\begin{array}{cc} & P_{i}=1,\\ & a_{i}=\rho_{i}^{2},\\ & b_{i}=\rho_{i},\\ & C_{i}=\frac{1}{1-\rho_{i}^{2}}, \end{array}\right\}$$

where *ρ*_*i*_ is now just the ordinary scalar correlation between the two outcome estimates in the *i*th study.

From equations (35), (37) and (38), the total variance contribution of the *i*th study is

$$\begin{array}{cc} T(V_{i}^{-1}) & =\frac{1}{s_{11}-s_{12}^{T}S_{22}^{-1}s_{12}}\left(1\;s_{12}^{T}S_{22}^{-1}\right)V_{i}^{-1}{\left(1\;s_{12}^{T}S_{22}^{-1}\right)}^{T}\\ & =\Omega_{11}(\frac{\sigma_{i}^{-2}}{1-a_{i}}-2\sigma_{i}^{-1}\frac{f_{i}^{T}b_{i}}{1-a_{i}}+f_{i}^{T}C_{i}f_{i})\;, \end{array}$$

where *f*_*i*_ is the (*p*−1)×1 vector

(40) $$f_{i}=\Lambda_{i}^{-1}S_{22}^{-1}s_{12}.$$

As before,

$$T\left(\sigma_{i}^{-2}11^{T}\right)=\Omega_{11}\sigma_{i}^{-2},$$

and so

$$B\left(V_{i}^{-1}\right)=T\left(V_{i}^{-1}\right)-T\left(\sigma_{i}^{-2}11^{T}\right)=\Omega_{11}(\sigma_{i}^{-2}\frac{a_{i}}{1-a_{i}}-2\sigma_{i}^{-1}\frac{f_{i}^{T}b_{i}}{1-a_{i}}+f_{i}^{T}C_{i}f_{i})\;.$$

This is a quadratic function of $\sigma_{i}^{-1}$: the accuracy of the primary outcome. Completing the square gives

(41) $$B\left(V_{i}^{-1}\right)=\Omega_{11}\left({\left[{\left(\frac{a_{i}}{1-a_{i}}\right)}^{1/2}\sigma_{i}^{-1}-\frac{f_{i}^{T}b_{i}}{{\left\{a_{i}\left(1-a_{i}\right)\right\}}^{1/2}}\right]}^{2}+f_{i}^{T}C_{i}f_{i}-\frac{{\left(f_{i}^{T}b_{i}\right)}^{2}}{a_{i}\left(1-a_{i}\right)}\right).$$

For a given meta‐analysis *s*_12_ and *S*_22_ are fixed, and so the vector *f*_*i*_ in equation (40) is just a linear function of the diagonal elements of $\Lambda_{i}^{-1}$: the accuracies of the secondary outcome estimates in the *i*th study.

For a simpler notation for equation (41), we extend the *u*_*i*_‐ and ${\hat{u}}_{i}$‐notation in the bivariate case to

(42) $$\begin{array}{cc} & u_{i}={\left(\frac{a_{i}}{1-a_{i}}\right)}^{1/2}\sigma_{i}^{-1},\\ & {\hat{u}}_{i}=\frac{f_{i}^{T}b_{i}}{{\left\{a_{i}\left(1-a_{i}\right)\right\}}^{1/2}}=s_{12}^{T}S_{22}^{-1}\frac{\Lambda_{i}^{-1}b_{i}}{{\left\{a_{i}\left(1-a_{i}\right)\right\}}^{1/2}}. \end{array}$$

For a given meta‐analysis (fixed values of *s*_11_,*s*_12_ and *S*_22_), *u*_*i*_ is proportional to the accuracy of the study's primary outcome and *v*_*i*_ is a scalar linear function of the accuracies of the secondary outcomes. If we define

$$g_{i}=\frac{{\left(f_{i}^{T}b_{i}\right)}^{2}}{a_{i}f^{T}P_{i}^{-1}f_{i}},$$

then, from expression (34),

$$f_{i}^{T}C_{i}f_{i}=f_{i}^{T}P_{i}^{-1}f_{i}+\frac{{\left(f_{i}^{T}b_{i}\right)}^{2}}{1-a_{i}}={\hat{u}}_{i}^{2}\left(\frac{1-a_{i}}{g_{i}}+a_{i}\right)\;\;,$$

from which we obtain

$$f_{i}^{T}C_{i}f_{i}-\frac{{\left(f_{i}^{T}b_{i}\right)}^{2}}{a_{i}\left(1-a_{i}\right)}=f_{i}^{T}C_{i}f_{i}-{\hat{u}}_{i}^{2}=\frac{\left(1-a_{i}\right)\left(1-g_{i}\right)}{g_{i}}{\hat{u}}_{i}^{2}.$$

Thus equation (41) is

(43) $$B\left(V_{i}^{-1}\right)=\Omega_{11}\left\{{\left(u_{i}-{\hat{u}}_{i}\right)}^{2}+\frac{\left(1-a_{i}\right)\left(1-g_{i}\right)}{g_{i}}{\hat{u}}_{i}^{2}\right\}\;\;,$$

and so

(44) $$T\left(V_{i}^{-1}\right)=\Omega_{11}\left\{\frac{1-a_{i}}{a_{i}}u_{i}^{2}+{\left(u_{i}-{\hat{u}}_{i}\right)}^{2}+\frac{\left(1-a_{i}\right)\left(1-g_{i}\right)}{g_{i}}{\hat{u}}_{i}^{2}\right\}\;\;.$$

Up to the scale factor $\Omega_{11}=\text{var}\left({\hat{\beta }}_{1}\right)$, equation (44) decomposes the total variance contribution of the *i*th study into three non‐negative parts, analogous to the main effects and interaction of the two factors *u*_*i*_ (proportional to the accuracy of the primary outcome) and ${\hat{u}}_{i}$ (a linear function of the accuracies of the secondary outcomes). The three effects are as follows:

(a)the term in $u_{i}^{2}$,
  $$\frac{1-a_{i}}{a_{i}}u_{i}^{2}=\sigma_{i}^{-2},$$
  the direct contribution of the primary outcome of the *i*th study as in univariate meta‐analysis;
(b)the term in ${\left(u_{i}-{\hat{u}}_{i}\right)}^{2}$ as in bivariate meta‐analysis, measuring the difference between the actual accuracy of the primary outcome and, in some sense, what might be expected from the pattern of *u*_*i*_s and ${\hat{u}}_{i}$s that is observed in the meta‐analysis as a whole;
(c)the term in ${\hat{u}}_{i}^{2}$, the additional effect of the accuracies of the study's secondary outcomes. This is 0 if *g*_*i*_=1.

The borrowing of strength for the *i*th study is proportional to the sum of the second and third terms of equation (44). The presence of the third term shows that there is a qualitative difference in borrowing‐of‐strength properties between the multivariate and bivariate cases. When *p*=2, the quantities *f*_*i*_, *b*_*i*_ and *P*_*i*_ are all scalars as in expression (39), and hence

$$g_{i}=\frac{{\left(f_{i}^{T}b_{i}\right)}^{2}}{a_{i}f_{i}^{T}P_{i}^{-1}f_{i}}=\frac{f_{i}^{2}b_{i}^{2}}{a_{i}f_{i}^{2}P_{i}^{-1}}=1.$$

Thus, when *p*=2, *g*_*i*_=1 for all *i* and so the third term in equation (44) is 0.

To see the equivalence of the ${\left(u_{i}-{\hat{u}}_{i}\right)}^{2}$‐term when *p*=2, the quantity ${\hat{u}}_{i}$ in equation (44), when expressed in the notation of Section 3.1, becomes

$${\hat{u}}_{i}=\frac{s_{12}}{{\left(1-\rho_{i}^{2}\right)}^{1/2}s_{22}}\nu_{i}^{-1},$$

which is just the same as equation (24). Hence, in the bivariate case, the residual $u_{i}-{\hat{u}}_{i}$ by using the definition in equation (42) is exactly the same as the residual $u_{i}-{\hat{u}}_{i}$ that was defined earlier in equation (25). The third term in equation (44) is still 0 in the multivariate case if *g*_*i*_=1, in which case the motivation of ${\hat{u}}_{i}$ as a least squares prediction of *u*_*i*_ continues to hold in the sense that $\Sigma u_{i}{\hat{u}}_{i}/\Sigma {\hat{u}}_{i}^{2}=1$.

### 4.2 Necessary and sufficient condition for no borrowing of strength

From $B(V_{i}^{-1})$ in equation (43), for there to be no borrowing of strength in the *i*th study we must have two conditions: *g*_*i*_=1 and $u_{i}={\hat{u}}_{i}$. We can exclude the trivial case *a*_*i*_=1 which would mean that there is an exact linear relationship between the *i*th study's primary and secondary outcome estimates.

For the first condition, *g*_*i*_=1 if *f*_*i*_=*kρ*_*i*_ for any arbitrary scalar factor *k*. This is also a necessary condition for *g*_*i*_=1, as can be verified directly by using a Lagrange multiplier calculation to find the maximum value of *g*_*i*_ for different values of *f*_*i*_. From equations (40) and (33), this means that

$$V_{i}=\left(\begin{array}{cc} \sigma_{i}^{2} & k^{-1}\sigma_{i}s_{12}^{T}S_{22}^{-1}\\ k^{-1}\sigma_{i}S_{22}^{-1}s_{12} & \Lambda_{i}P_{i}\Lambda_{i} \end{array}\right)\;\;.$$

Comparing this with equation (37), the equivalent condition is that the covariance vector part of *V*_*i*_ in equation (33) is a scalar multiple of the corresponding covariance vector part of Ω (or of $\overset{¯}{V}$).

For the second condition, if *f*_*i*_=*kρ*_*i*_ then

$${\hat{u}}_{i}=\frac{f_{i}^{T}b_{i}}{{\left\{a_{i}\left(1-a_{i}\right)\right\}}^{1/2}}=\frac{k\rho_{i}^{T}P_{i}^{-1}\rho_{i}}{{\left\{a_{i}\left(1-a_{i}\right)\right\}}^{1/2}}=k\;\;{\left(\frac{a_{i}}{1-a_{i}}\right)}^{1/2}\;\;,$$

and so

$${\left(u_{i}-{\hat{u}}_{i}\right)}^{2}=\frac{a_{i}}{1-a^{i}}{\left(\sigma_{i}^{-1}-k\right)}^{2}.$$

So if $u_{i}={\hat{u}}_{i}$ then $k=\sigma_{i}^{-1}$. This extends the proportionality between the covariance vector parts of *V*_*i*_ and Ω required for *g*_*i*_=1 to include also the (1,1) term. So the necessary and sufficient condition for no borrowing of strength is that the first row (or first column) of *V*_*i*_ must be a scalar multiple of the corresponding row or column of the harmonic mean matrix $\overset{¯}{V}$. Thus the necessary and sufficient condition (29) in the bivariate case generalizes directly to the multivariate case, where *V*_*i*12_ and ${\overset{¯}{V}}_{12}$ are now the covariance vector components of *V*_*i*_ and $\overset{¯}{V}$ respectively.

Note that condition (29) gives no constraint on the size of the scalar multiple *k* involved, and hence no constraint on the sample size of the trial. Both small and large trials may end up giving no borrowing of strength, including studies with large correlations between the primary and secondary outcomes. Note also that condition (29) imposes no constraint on the (2,2) partition of *V* in equation (33), i.e. on the distribution of the estimates for the secondary outcomes *per se*.

We commented in Section 4.1 that the some of the components of $V_{i}^{-1}$ in equation (35) can be interpreted in terms of a within‐study multiple regression of *y*_*i*1_ on *y*_*i*2_. We can similarly interpret equation (29) in terms of the regression the other way round, predicting the vector of secondary outcome estimates *y*_2*i*_ from the primary outcome estimate *y*_1*i*_. The vector of regression coefficients for the *i*th study would then be *V*_*i*12_/*V*_*i*11_, which is just the left‐hand side of equation (29). Hence the necessary and sufficient condition for the *i*th study to give no borrowing of strength is that the within‐study vector of regression coefficients for predicting the secondary from the primary estimates is the same as the corresponding regression vector for a study with the harmonic mean variance matrix $\overset{¯}{V}$.

## 5 Random‐effects models

The results in this paper depend on some important assumptions, most obviously the assumption of a fixed effects model, that all studies are modelled by equation (1). This strong assumption, that all the studies are estimating the same treatment effect *β*, has been widely discussed in the univariate meta‐analysis literature. Jackson *et al*. (2017) followed some other references on multivariate meta‐analysis by also including random‐effects models. These references generalize the usual two‐stage approach to univariate random‐effects meta‐analysis by first estimating a between‐studies variance matrix Ψ by $\hat{\Psi }$ and then using the fixed effects model (1) with each *V*_*i*_ replaced by $V_{i}+\hat{\Psi }$. Jackson *et al*. (2010) showed how the familiar univariate DL estimate (DerSimonian and Laird, 1986) can be extended to the multivariate case, using the univariate DL estimates for each outcome taken individually, and analogous method‐of‐moments estimates for each covariance component. Other methods of estimating Ψ have been discussed in several recent references (Chen *et al*., 2012; Jackson *et al*., 2013; Ma and Mazumdar, 2011).

The borrowing‐of‐strength quantities *E* and $B_{i}=B\left(V_{i}^{-1}\right)$ that were discussed earlier are descriptive measures of how much the multivariate estimation of *β*_1_ has been influenced by the data on the secondary outcomes. In random‐effects models, the corresponding estimates $\hat{E}$ and ${\hat{B}}_{i}=B\left({\hat{V}}_{\mathrm{RE}i}^{-1}\right)$ calculated from the fitted marginal variance matrices ${\hat{V}}_{\mathrm{RE}i}=V_{i}+\hat{\Psi }$ are similarly descriptive measures of the role of the secondary outcomes within the fitted model. The definition of *E* in equation (5) is only a valid measure of efficiency if the variances of the two estimates being compared are based on a consistent model, which means that the diagonal element of $\hat{\Psi }$ for the primary outcome must be the same as the univariate random‐effects variance estimate that we would obtain if we fitted a univariate random‐effects meta‐analysis model to the data on the primary outcome alone. Only under this condition do we retain the same interpretation of $\hat{E}$ and ${\hat{B}}_{i}$ as discussed earlier for fixed effects models. In practice, DL estimates are almost always used in univariate random‐effects meta‐analysis, suggesting that $\hat{\Psi }$ should be estimated by using a method‐of‐moments estimate which retains the univariate DL estimates as its diagonal elements. A slight modification to the truncation step in Jackson *et al*. (2010) is needed to ensure that this is always so, which for bivariate meta‐analysis (as in the example below) simply amounts to truncating the estimated random‐effects correlation to its nearest value in the interval [−1,1]. We can then retain the same interpretation of $\hat{E}$ and ${\hat{B}}_{i}$ as a direct comparison of the fitted variance of ${\hat{\beta }}_{1}$ by using all of the data with the fitted variance that we would obtain from a univariate meta‐analysis using only the data on the primary outcomes. In this sense, the theory and interpretation of borrowing‐of‐strength statistics for fixed effects models applies in exactly the same way to random‐effects models, as implied by the discussion in Jackson *et al*. (2017), section 4.

As the variance matrices *V*_*i*_ are assumed known, the descriptive measures *E* and *B*_*i*_ can also be given an inferential interpretation as estimates of the borrowing‐of‐strength parameters of the true underlying model (1). However, applying this to random‐effects models raises different issues, since now the marginal variance matrices ${\hat{V}}_{\mathrm{RE}i}$ depend on $\hat{\Psi }$, which can exhibit substantial sampling uncertainty if *n* is small (Guolo and Varin, 2017). Arguably, $\hat{\Psi }$ has a greater influence on $\hat{E}$ and on ${\hat{B}}_{i}$ than it has on the more usual problem of estimating *β*, since $\hat{\beta }$ retains its unbiasedness property conditionally on all possible values of $\hat{\Psi }$. However, the example below suggests that $\hat{E}$ and ${\hat{B}}_{i}$ can still provide useful estimates of the borrowing‐of‐strength properties of the true underlying random‐effects model.

As a simple illustration in the bivariate case, suppose that the treatment effect estimates in the example of Section 3.2 were in fact generated from the bivariate random‐effects model

(45) $$y_{i}∼N\left(\beta ,V_{i}+\Psi \right),i=1,2,\ldots ,10,$$

with $\Psi =\alpha \overset{¯}{V}$ and *α*⩾0, where the *V*_*i*_s are as in Table 1 and $\overset{¯}{V}$ is their harmonic mean as in equation (4). Then by increasing *α* from 0 (the fixed effects model) we obtain increasing between‐study heterogeneity. A small value of *α* means that the fixed effects model slightly underestimates the variability of the *y*_*i*_s, and the assumed form of Ψ means that the pattern of variances remains reasonably similar to those observed in the data. We can then simulate vectors *y*_*i*_ from equation (45) and compare the borrowing‐of‐strength statistics that are calculated from the actual marginal variance matrices $V_{\mathrm{RE}i}=V_{i}+\alpha \overset{¯}{V}$ with the corresponding statistics calculated from the estimated marginal variances ${\hat{V}}_{\mathrm{RE}i}=V_{i}+\hat{\Psi }$. For the reason discussed above, we calculate $\hat{\Psi }$ by using the slightly modified version of the method of Jackson *et al*. (2010) which was mentioned earlier.

Table 2 describes the results of a small simulation study based on 1000 replications for each of five values of *α*, ranging from *α*=0 (fixed effects) to *α*=2 (quite substantial heterogeneity). We assume that the primary interest is the value of *β*_2_: the log‐hazard‐ratio for the risk of stroke. The second column of Table 2 shows the actual efficiencies *E* based on *V*_RE*i*_. As expected, the entry 0.888 for *α*=0 is just the fixed effects efficiency that has already been quoted in Section 3.2. Adding the same variance matrix to each *V*_*i*_ has the effect of reducing the relative differences between them, which explains why the values of *E* tend to increase as *α* increases. The estimated efficiencies $\hat{E}$ based on ${\hat{V}}_{\mathrm{RE}i}$ vary randomly between simulations, but their sample medians across the 1000 simulations, which are shown in the third column of Table 2, also follow a similar pattern. We summarize the simulation results by using medians rather than means because of skewness that is caused by the truncation of DL estimates. Section 2.1 has shown that the actual study‐specific borrowing‐of‐strength components *B*_*i*_ always add up to 1−*E* and so when, in the random‐effects model, $\hat{E}$ is different from *E* we cannot expect the corresponding estimated and true borrowing‐of‐strength components to be exactly comparable. However, from a practical point of view, what we would hope to see is that the studies which show the greatest (or least) borrowing of strength under the estimated model are the same, or substantially the same, as the studies which give the greatest (or least) borrowing of strength under the true model. For each simulation, the extent to which this is so is measured by the rank correlation $\text{rc}({\hat{B}}_{i},B_{i})$. The fourth column of Table 2 shows the sample medians of these rank correlations. These are satisfactorily high (90% or greater) for the smaller values of *α* but, as expected, tend to deteriorate slightly as the heterogeneity increases.

**Table 2:** Simulation of a random‐effects variant of the example for estimating *β*_2_, comparing the estimated efficiency and borrowing of strength for the fitted random‐effects model with their corresponding true values†

*α*	*True efficiency*	*Median*	*Median*
	*E*_RE_	${\hat{E}}_{\mathrm{RE}}$	*rc*(*BoS*_*i*_, ${\hat{\mathrm{BoS}}}_{i})$
0	0.888	0.903	0.927
0.5	0.932	0.928	0.903
1	0.948	0.945	0.891
1.5	0.957	0.947	0.867
2	0.961	0.954	0.842

†Increasing values of *α* indicate increasing heterogeneity.

## 6 Discussion

In most statistical problems, taking into account data on relevant covariates or confounders leads to more accurate estimates and predictions, especially if the secondary variables are closely correlated with the main variable of interest. However, this is not necessarily so in meta‐analysis—multivariate meta‐analysis can give little or no improvement over univariate methods even if the secondary outcomes are closely correlated with the primary outcome. By writing the borrowing‐of‐strength measure ${\text{BoS}}_{i1}^{\mathrm{SD}}$ that was proposed by Jackson *et al*. (2017) as the explicit function *B*(*V* ^−1^) in equation (11), and then evaluating some of this function's mathematical properties, we have shed light on how and why individual study characteristics may or may not lead to a useful role for secondary outcomes in multivariate meta‐analysis.

The paper has made some important assumptions. The fixed effects model (1) and its application to random‐effects models has been discussed in Section 5. We have also assumed that, by replacing a within‐study inverse variance *V* ^−1^ by $V_{*}^{-1}$ in equation (17), the fixed effects formulae continue to apply when one or more of the outcomes is missing. This is only valid under the assumption of data missing at random, that the chance of an outcome being unreported can be modelled as an independent chance mechanism conditional on the outcome estimates which actually are observed. Acknowledging this assumption can be crucially important in meta‐analysis, where outcome reporting bias, e.g. when several outcomes are measured but only those showing a statistically significant effect are reported, is a common problem (Kirkham *et al*., 2010), although the simulations in Kirkham *et al*. (2012) suggest that in some circumstances multivariate methods can be more robust than univariate methods to departures from this assumption. Subjective assessments of the risk of outcome reporting bias (Kirkham *et al*., 2010) can lead to useful univariate bias corrections (Copas *et al*., 2013), and an extension to multivariate models may also be possible.

The paper has also assumed that the *V*_*i*_s (or the $V_{*i}^{-1}$s) are known, so that borrowing‐of‐strength measures can be evaluated explicitly. Riley (2009) emphasized the importance of taking the within‐study correlations into account and discussed the problem when, in practice, authors of research papers may report only estimates and standard errors for the outcomes taken one at a time. In that case only the diagonal elements of *V*_*i*_ are provided directly in study reports. If we can obtain full data (individual patient data) for such studies, then consistent estimates of the within‐study correlations can be calculated, but in practice this may be difficult or impossible. Various approaches to dealing with this issue have been suggested, such as sensitivity analyses that explore a variety of within‐study correlations (Jackson *et al*., 2011). The special case of equal within‐study correlations (Section 3.3) can be a useful starting point. Wei and Higgins (2013) examined ways in which it may be possible to estimate these correlations retrospectively from other information that might be available. A partial approach is to note our finding that most borrowing of strength comes from studies whose designs are most atypical of the studies as a whole, and so by comparing the research methods that are used in the studies it may be possible at least to identify roughly which studies might be worth following up. Concentrating on trying to obtain further data for just some of these studies, and using the missing data formula (17) for other studies, may give at least some indication of whether including secondary outcomes in a multivariate model offers the potential to improve the estimate of the primary treatment effect.

In practice the matrix *V*_*i*_ is calculated from the data in the *i*th study, and so the assumption that the *V*_*i*_s are known is ignoring the sampling error in these variance estimates. Table 1 shows that the example in Section 3.2 is based on large sample sizes, but with smaller samples the resulting inferences can underestimate uncertainty and be biased in cases where the estimated variances are correlated with the values of the *y*s. In univariate meta‐analysis this bias is particularly noticeable in the Egger test for funnel plot symmetry (Egger *et al*., 1997), as demonstrated in several simulation studies. Copas and Lozada‐Can (2009) gave a general method for calculating bias corrections for such test statistics. Berkey *et al*.(1995) suggested a simpler way of eliminating bias, by smoothing the variance estimates across the studies. Assuming that study variances are inversely proportional to study sample size, and estimating the proportionality factor from the studies as a whole, essentially eliminates the correlation between the outcomes and their variances. Harbord *et al*. (2006) suggested a similar idea. However, for estimating efficiency and borrowing of strength as discussed in this paper, such considerations of bias are not directly relevant as *E* and $B(V_{i}^{-1})$ depend only on the *V*_*i*_s and not on the actual values of the *y*_*i*_s. If each estimated *V*_*i*_ is consistent then so will be the estimates of the borrowing‐of‐strength quantities derived. It is important to avoid any smoothing of the *V*_*i*_s so that they properly reflect the characteristics of each individual study.
